# Protein Tyrosine Phosphatase-Induced Hyperactivity Is a Conserved Strategy of a Subset of BaculoViruses to Manipulate Lepidopteran Host Behavior

**DOI:** 10.1371/journal.pone.0046933

**Published:** 2012-10-15

**Authors:** Stineke van Houte, Vera I. D. Ros, Tom G. Mastenbroek, Nadia J. Vendrig, Kelli Hoover, Jeroen Spitzen, Monique M. van Oers

**Affiliations:** 1 Laboratory of Virology, Wageningen University, Wageningen, The Netherlands; 2 Biometris, Wageningen University, Wageningen, The Netherlands; 3 Department of Entomology and Center for Chemical Ecology, Pennsylvania State University, University Park, Pennsylvania, United States of America; 4 Laboratory of Entomology, Wageningen University, Wageningen, The Netherlands; Oxford Brookes University, United Kingdom

## Abstract

Many parasites manipulate host behavior to increase the probability of transmission. To date, direct evidence for parasitic genes underlying such behavioral manipulations is scarce. Here we show that the baculovirus *Autographa californica* nuclear polyhedrovirus (AcMNPV) induces hyperactive behavior in *Spodoptera exigua* larvae at three days after infection. Furthermore, we identify the viral protein tyrosine phosphatase (*ptp*) gene as a key player in the induction of hyperactivity in larvae, and show that mutating the catalytic site of the encoded phosphatase enzyme prevents this induced behavior. Phylogenetic inference points at a lepidopteran origin of the *ptp* gene and shows that this gene is well-conserved in a group of related baculoviruses. Our study suggests that *ptp*-induced behavioral manipulation is an evolutionarily conserved strategy of this group of baculoviruses to enhance virus transmission, and represents an example of the extended phenotype concept. Overall, these data provide a firm base for a deeper understanding of the mechanisms behind baculovirus-induced insect behavior.

## Introduction

Modification of host behavior upon parasitic infection is an intriguing phenomenon that has been observed in a wide range of organisms [1], [2], [3]. Parasites can affect a large variety of behavioral traits, including feeding behavior, mating, odor response, and locomotor activity [2]. Many of these behavioral changes are thought to represent manipulative strategies of the parasite to increase parasite transmission rates. As such, the altered behavior of the host is regulated by the expression of parasitic genes, thus representing the parasite's extended phenotype [4].

Aquatic isopods (*Asellus intermedius*), for example, display hyperactive behavior when they are infected with the parasitic worm *Acanthocephalus dirus* [5]. This behavioral change is thought to make the isopods more susceptible to predation by fish, which serve as a final host for the acanthocephalan worm [6]. Also arthropod-borne parasites may alter the behavior of their hosts to increase their chances of transmission [7], [8], [9]. *Aedes aegypti* mosquitoes carrying Dengue virus (*Flaviviridae*) display a 50% higher locomotor activity than uninfected mosquitoes [10], and show a longer probing and feeding time [11], thereby potentially increasing the transmission rates of the virus. Although numerous examples of such manipulations are known, direct evidence for parasitic genes underlying these phenomena is scarce and the molecular mechanisms by which such genes alter behavior are still largely enigmatic [3], [12], [13].

Baculoviruses are known to induce behavioral changes in their caterpillar hosts, consisting of abnormal climbing behavior, named ‘Wipfelkrankheit’ or ‘tree top disease’, and hyperactivity [14], [15]. Both behavioral changes are thought to enhance the spread of progeny virions released from dead and liquefied caterpillars over a large surface of plant foliage, thus increasing transmission rates to subsequent generations of caterpillar hosts. Although the molecular mechanisms underlying baculovirus-induced behavior are still largely unknown, recent work provided some first clues about two baculoviral genes involved in either one of these extended phenotypes. Firstly, the ecdysteroid uridine 5′-diphosphate (UDP)–glucosyltransferase (*egt*) gene of *Lymantria dispar* multiple nucleopolyhedrovirus (LdMNPV) was found to induce climbing behavior in the European gypsy moth *L. dispar* [16], resulting in tree top disease. On the other hand, Kamita *et al.* (2005) and Katsuma *et al.* (2012) showed the involvement of the *Bombyx mori* nucleopolyhedrovirus (BmNPV) *ptp* gene, encoding a protein tyrosine phosphatase, in the induction of enhanced locomotor activity in larvae of the silkmoth *B. mori* [17], [18]. However, at present it is unclear how well-conserved the *ptp* gene is among the members of the family *Baculoviridae*, and whether this *ptp*-induced behavior reflects an evolutionary conserved strategy of baculoviruses to manipulate their insect host. Moreover, notwithstanding the considerable advances that the study by Kamita *et al.* (2005) has made in understanding the molecular basis of this parasite-induced hyperactive extended phenotype, it is unknown to what extent this particular pathogen-host system reflects ecologically meaningful interactions between baculoviruses and lepidopteran hosts. This is a relevant question, as since the foundation of sericulture, approximately 5000 years ago, *B. mori* became a fully domesticated insect that has undergone extensive inbreeding and artificial selection, mainly for silk production optimization purposes [19]. During this selection process *B. mori* lost several typical behavioral traits, including flight and predator and disease avoidance, whereas it gained other characteristics, *e.g.*, increased tolerance to larval crowding [19]. Gain or loss of such traits can be expected to strongly affect phenomena such as parasitic manipulation of behavior. This is illustrated by the fact that the *B. mori* strain used in the locomotor activity assays showed exceptionally low endogenous activity levels in the absence of virus infection [17], while the wild silkmoth *B. mandarina*, which is considered the ancestral species from which *B. mori* originated [20], is known to display relatively high activity levels in the field [21]. Therefore, it is important to understand whether a possible virus-induced behavioral manipulation is conserved in insect species that did not undergo such extensive artificial selection, and presumably have evolved strategies to counteract parasitic manipulation. Behavioral alterations observed in such insects are more likely to reflect a natural situation in which the manipulation is the result of a long coevolutionary history between parasite and host.

A homolog of the BmNPV *ptp* gene is present in the baculovirus type species *Autographa californica* multiple nucleopolyhedrovirus (AcMNPV) [22]. Previous work on this AcMNPV *ptp* gene has shown that it is not essential for virus replication [23]. The enzyme encoded by this gene contains the HC signature motif that is characteristic for all members of the PTP protein family (reviewed in [24]), and dephosphorylates both RNA [25], [26] and protein substrates [27] *in vitro*. Mutating the catalytic Cys-119 residue in the HC motif to either alanine (C119A) or serine (C119S) was shown to abolish enzymatic activity of the AcMNPV PTP protein [25], [26], [27].

To examine whether baculoviral *ptp*-induced behavior reflects an evolutionary conserved strategy, we used AcMNPV and its lepidopteran host *Spodoptera exigua* (Hübner) (Lepidoptera, Noctuidae) as a model system for studying behavioral manipulation and its underlying mechanisms. AcMNPV represents the best characterized baculovirus to date, for which well-developed genetic tools exist [28]. *Spodoptera exigua* or the beet armyworm is a polyphagous insect pest species that occurs worldwide in tropical and subtropical areas. Although AcMNPV has a broad host range, with over 32 insect species known to be susceptible to the virus [29], *S. exigua* was chosen as a host model, as the virus is highly infectious to this natural host. This combination of virus and host is relatively commonly used as *S. exigua* can be easily reared under laboratory conditions.

We show that AcMNPV induces hyperactivity in *S. exigua* larvae at three days after infection. Subsequently, the possible involvement of the AcMNPV *ptp* gene in this behavioral change was studied, and we analyzed whether the phosphatase activity of its encoded enzyme is required for this behavioral change. Phylogenetic analyses were performed to gain insight into the origin of the baculovirus *ptp* gene and to determine its degree of conservation within the family *Baculoviridae*. Our results strongly suggest that *ptp*-mediated manipulation of behavior represents an evolutionarily conserved strategy among a subset of baculoviruses. Overall, these findings contribute to a better understanding of the mechanisms governing parasite-induced behavioral changes.

## Materials and Methods

### Insect cells and larvae

*Spodoptera frugiperda* 9 (Sf9) cells (Invitrogen) were cultured as monolayers in Sf900II serum-free medium (Invitrogen) supplemented with 5% fetal bovine serum (Invitrogen) and 0.1% gentamycin (Invitrogen). *Spodoptera exigua* larvae were reared on artificial diet at 27°C with 50% relative humidity as described before [30], and a 14∶10 h light∶dark photoperiod.

### Generation of recombinant bacmids

**Figure 1:**
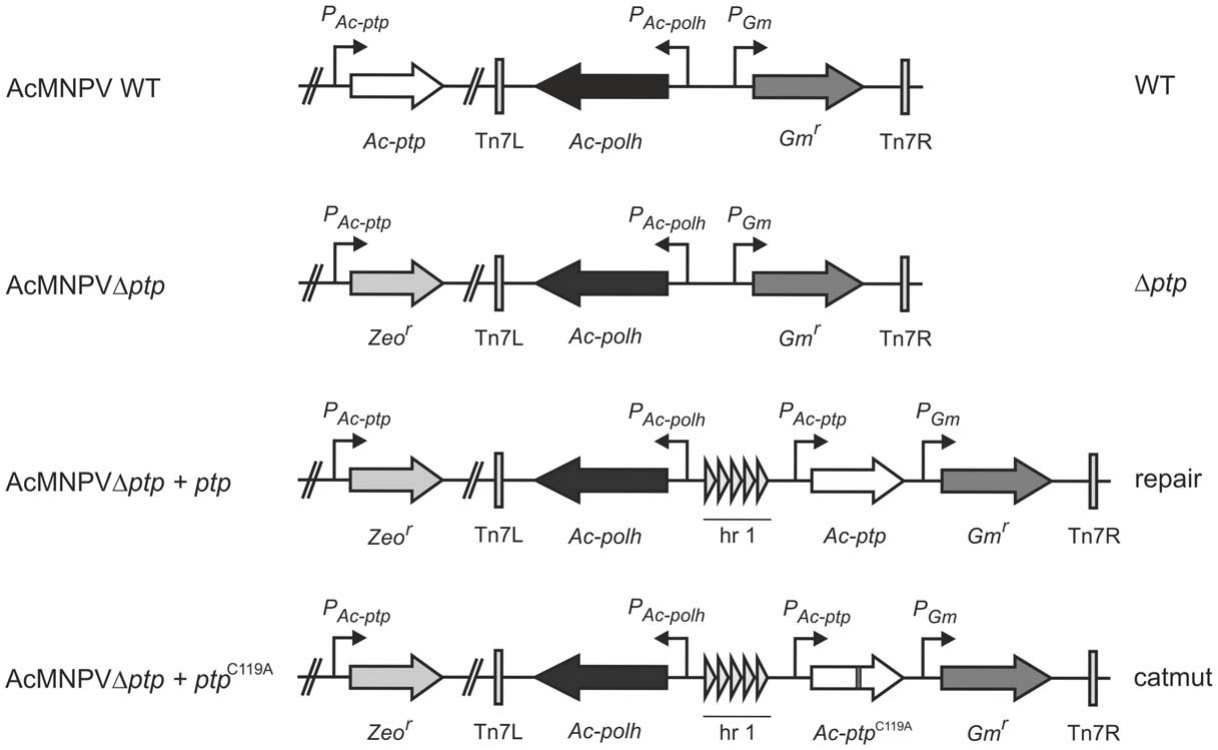
Overview of the recombinant bacmids used in this study. On the left the names of the recombinant bacmids are indicated, while on the right the corresponding abbreviated names as they are used in this paper are shown.

The AcMNPV E2 bacmid was used as wild type (WT) virus in this study [31]. An AcMNPV bacmid with a deletion of the *ptp* gene (Δ*ptp*), derived from this WT bacmid, was kindly provided by Linda Guarino of Texas A&M University, USA [32]. The Δ*ptp* virus was originally created by replacing nucleotide (nt) positions 509 to 1080, a region spanning the complete *ptp* ORF, with a Zeocin resistance marker gene [32]. To enable oral infection of larvae, the open reading frame (ORF) of the AcMNPV polyhedrin (*polh*) gene was placed back into the WT and Δ*ptp* genomes. For this purpose, Bac-to-Bac transposition [28] was performed with a modified pFastBacDual vector (pFBDpolhΔp10), in which the AcMNPV *polh* ORF was cloned downstream of the *polh* promoter, and from which the *p10* promoter was removed, both as described in [33] (Fig. 1). To ensure that a possible phenotype of the Δ*ptp* recombinant virus was not due to any other genome mutations, a repair bacmid was created for which the Δ*ptp* bacmid was used as a backbone. On the AcMNPV genome [22] the *homologous repeat region* (*hr*) *1* sequence is mapped at nt positions 133883 to 133894 and 1 to 445, ending 48 nt upstream of the *ptp* ORF (which is AcORF1). As baculovirus *hrs* are known to be involved in enhancing the expression of downstream genes [34], the repair bacmid was generated in such a way that the *ptp* gene was placed back together with the upstream *hr1* sequence. The coding sequence of the *ptp* ORF and the upstream 532 base pairs (bp), containing the *hr1* repeat region and the putative *ptp* promoter sequence [35], were PCR amplified with the proofreading polymerase Phusion (Finnzymes), using primer 1 combined with primer 2 (Table S1). To allow subsequent cloning, *Nco*I and *Nsi*I restriction sites (underlined in the primer sequence, Table S1) were introduced with these primers. The sequenced PCR product was cloned as *Nco*I/*Nsi*I fragment into pFBDpolhΔp10. Subsequently, the transposon carrying the *ptp* and *polh* sequences was introduced into the Δ*ptp* bacmid as described above (Fig. 1).

For construction of a recombinant bacmid encoding a catalytically inactive PTP protein (catmut), a mutation was introduced in the HC signature motif. The Cys-119 residue was replaced with an alanine (C119A) by overlap extension PCR with Phusion polymerase, using primer 3, which introduced two point mutations at nt 355 and 356 relative to the ATG start codon, combined with primer 2 (Table S1). The resulting 241 bp product was then used as reverse primer combined with primer 1, thus generating the *ptp* ^C119A^ ORF with the upstream *hr1* sequence and putative promoter sequence. This sequenced fragment was cloned as *Nco*I/*Nsi*I fragment into the pFBDpolhΔp10 vector, and inserted into the Δ*ptp* bacmid as described above (Fig. 1).

### Amplification and purification of virus

To produce recombinant viruses, Sf9 cells were transfected with the above described recombinant bacmids (WT, Δ*ptp*, repair and catmut) using CellFectin II transfection reagent (Invitrogen), and the resulting budded virus (BV) stock was amplified once in cells. The viral occlusion bodies (OBs) generated in these infected cells were amplified in *S. exigua* third (L3) and fourth (L4) larval instars. Purification of OBs from infected larvae was done by grinding deceased larvae in water and filtering through a double layer of cheese cloth. The suspension was first centrifuged at 500×*g*, after which the supernatant was centrifuged at 4000×*g* to pellet OBs. Finally, OBs were resuspended in water and stored at 4°C.

### Infectivity assays

Infectivity assays were performed to determine the 50% lethal virus concentration (LC_50_) for each virus. *Spodoptera exigua* larvae were grown until the late L2 stage. Larvae were starved overnight for 16 h and allowed to molt during that time. Newly molted L3 larvae were selected and infected using droplet feeding. Dilutions of viral OB stocks were prepared in a 10% sucrose solution containing 0.4% (w/v) Patent Blue V food coloring dye (Sigma-Aldrich). For each virus (WT, Δ*ptp*, repair, and catmut) the following dilutions were prepared: 10^4^, 10^5^, 10^6^, 10^7^, 10^8^ and 10^9^ OBs/ml. For each dilution, 24–36 larvae were allowed to drink from the virus suspension for 15 minutes. Mock-infected larvae were used as controls and were droplet fed using a virus-free 10% sucrose solution containing 0.4% (w/v) Patent Blue V food coloring dye. Only larvae with a completely blue-colored gut were selected, as these were assumed to have ingested an equal volume of virus suspension. These larvae were reared individually on artificial diet in 12-well plates. Once a day, larvae were scored for mortality until all larvae were either dead or had pupated. To determine the 50% lethal time (LT_50_) values for each of the viruses, larvae infected with an LC_90–95_ dose (10^8^ OBs/ml) were checked twice a day for mortality until all larvae were dead or had pupated (mock). Median LC_50_ values were determined by Probit analysis, and median LT_50_ values were determined using Kaplan-Meier survival analysis. Both analyses were performed using SPSS 19.0.

### Movement assays

Late L2 larvae were starved for 16 h and allowed to molt as described above. Newly molted L3 larvae were infected with an LC_90–95_ dose (10^8^ OBs/ml) of virus using droplet feeding, and subsequently fed on artificial diet. To measure larval activity, individual larvae were placed in an arena consisting of a plain surface with dimensions of 120×90 cm, which was equipped with a digital video camera (Sony) positioned at 200 cm above the arena surface. The camera was flanked by two photography studio lights of 40 W each to ensure an equal light distribution throughout the arena surface. The temperature at which the measurements were done was 24.5°C±0.7. While being recorded, each larva was allowed to move freely for 10 minutes in the arena. To calculate the distance (i.e. locomotor activity) travelled by individual larvae, digital recordings were analyzed using EthoVision tracking software (Noldus Information Technology, The Netherlands). This is a commonly used software analysis method for different types of behavioral assays (for a review see [36]).

In the first experiment, the distance moved by mock- and WT-infected larvae was measured at one, two and three days post-infection (dpi). Two replicates were performed, and data were analyzed using a linear mixed model (Proc Mixed procedure, SAS Inst. Inc., 2004) with treatment, days post-infection, experiment number, and their two-way interactions as fixed effects, days post-infection as within-subject repeated measures, and an unstructured covariance structure. The full-model was reduced using backward stepwise elimination of fixed effects based on Kenward-Rogers approximate F-test (α>0.05), and least square means differences for treatment, days post-infection and their interaction were then tested for significance using Bonferroni-adjusted P-values.

In the second experiment, distance moved by mock-, WT-, Δ*ptp-*, repair-, and catmut-infected larvae was measured at three dpi. For statistical analysis of the data, distances travelled were normalized using a Box-Cox transformation [37]. In a single movement assay two or three treatments were measured, one always being the WT treatment, so that it could be used as an internal standard for the different assays performed. Two or three replicates were performed per treatment. The relation between distance travelled at three days post-infection and treatment (mock, WT, Δ*ptp*, repair and catmut) was analyzed using general linear models (Proc GLM procedure, SAS Inst. Inc., 2004) with treatment and experiment number, and their interaction, as fixed effects. The full-model was reduced using backward stepwise elimination of fixed effects based on the F-test (α>0.05) with type III sum of squares, and least square means differences of treatments were then tested for significance using Bonferroni-adjusted P-values.

### RNA isolation and RT-PCR

Newly molted L3 larvae were infected by droplet feeding with an LC_90–95_ dose of virus as described above. At three dpi total RNA from single larvae was extracted by homogenizing in 250 µl Trizol reagent (Invitrogen) and total RNA was isolated following the manufacturer's instructions. The RNA pellet was dissolved in 50 µl water and heated for 10 min at 55°C. Any contaminating DNA was removed with the DNA*free* kit (Applied Biosystems) according to the company's protocol. Production of cDNA was performed using SuperScript III Reverse Transcriptase (Invitrogen) according to the company's protocol. RT-PCR was performed using primers to amplify i) 426 bp within the AcMNPV *ptp* ORF (primers 5 and 6, Table S1), ii) 512 bp within the AcMNPV *ie1* ORF (primers 7 and 8), and iii) 486 bp of the *S. exigua* host *Se-eIF5A* ORF (primers 9 and 10). For each RT sample, a control sample was run in which the RT step was omitted (non-RT) to check for DNA contamination. In addition, a negative control without template was processed for each primer pair.

### Phylogenetic analysis

GenBank and Butterflybase/InsectaCentral [38] were explored to obtain *ptp* nucleotide sequences (Table S2). A homologous *ptp* sequence from a *S. exigua* EST bank was provided by Salva Herrero, Universitat de València, Spain [39]. BLAST (National Center for Biotechnology Information) was used for nucleotide and predicted amino acid sequence homology searches. Multiple searches were performed, using different baculovirus and lepidopteran *ptp* sequences as a query. All known invertebrate (20), baculovirus (15), and poxvirus (2) *ptp* genes were included in the analysis. Sequences were translated in frame to proteins and aligned using MAFFT version 6 with default settings [40]. Protein alignment was converted back into the corresponding codon alignment using PAL2NAL [41]. Gblocks [42] was used for trimming sequences to select conserved domains. PAUP* version 4.0b10 [43] was used to select the optimal evolution model, as described in [44]. ML analysis (heuristic search, 100 bootstrap replicates) was performed in PAUP, using a submodel of the General Time Reversible Model with invariable sites and a gamma distribution of rate heterogeneity (GTR+I+G) with rate class ‘abcdec’. Bayesian inference was conducted using MRBAYES 3.1.2 [45], using the GTR+I+G model (default settings, six million generations, burn-in of 25%).

A *lef-8* phylogeny was also constructed as described above (model: GTR+I+G, rate class: ‘abccde’) and includes the *lef-8* sequences of all baculoviruses that have a *ptp* and/or *ptp2* gene (except for *Iragoides fasciata* NPV for which the *lef-8* sequence is not available in GenBank). In addition, we included all Alpha- and Betabaculoviruses for which the genome sequence is available in GenBank and for which the absence of both *ptp* and *ptp2* genes was confirmed (Table S3).

## Results

### Larval infectivity is not affected by deletion of the AcMNPV ptp gene

To investigate whether AcMNPV induces altered behavior in the permissive host *S. exigua*, and to determine whether the viral *ptp* gene plays a role in this, we performed behavioral studies using four different viruses: WT, Δ*ptp*, repair and catmut (Fig. 1). First, the lethal concentration (LC_50_) and lethal time (LT_50_) of these virus stocks were determined in *S. exigua* L3 larvae. The LC_50_ values for WT, Δ*ptp*, repair, and catmut were 10^6.4^, 10^6.5^, 10^6.6^, and 10^6.7^ OBs/ml, respectively (Table 1), and overlapping 95% fiducial limits indicated that these values were not significantly different. Likewise, upon infection with an LC_90–95_ viral dose, no significant differences in the time to death were found among the viruses, with LT_50_ values of 112, 120, 100, and 112 h, respectively (Table 1) (χ^2^ = 1.291, df = 3, P = 0.731), although the 95% fiducial limits interval of the LT_50_ value of AcMNPV Δ*ptp* was quite large (38 h). It is possible that differences in LT_50_ may exist upon infection with lower viral doses, although this would not be relevant for this study as only LC_90–95_ viral doses were used in the movement assays. Overall, these indicates that viral infectivity and speed of kill are not affected by the absence of the *ptp* gene (Δ*ptp*) or the presence of a mutant *ptp* gene (catmut) during infection of *S. exigua* larvae. Li and Miller (1995) reported that upon amplification in Sf21 insect cells, the AcMNPV Δ*ptp* virus infectivity was reduced 50% compared to a WT strain [23]. However, they also showed that this potency difference disappeared upon oral infection in *S. frugiperda* larvae, and that the lethal concentration and lethal time were similar between WT and Δ*ptp* virus, which is in correspondence with our findings for *S. exigua*.

**Table 1:** Dose-mortality response (log LC_50_) and time-mortality response (LT_50_) of L3 *S. exigua* larvae infected with WT, Δ*ptp*, repair and catmut virus. LT_50_ determined for a virus concentration of 10^8^ OBs/ml (∼90–95% mortality).

		log 95% fiducial limits (OBs/ml)			95% fiducial limits (h)	
Virus	log LC_50_ (OBs/ml)	*lower*	*upper*	LT_50_ (h)	*lower*	*upper*
WT	6.4	6.1	6.8	112	97	127
Δ*ptp*	6.5	6.2	6.8	120	101	139
repair	6.6	6.3	6.9	100	92	108
catmut	6.7	6.4	7.0	112	104	120

### AcMNPV induces hyperactive behavior in its host S. exigua

**Figure 2:**
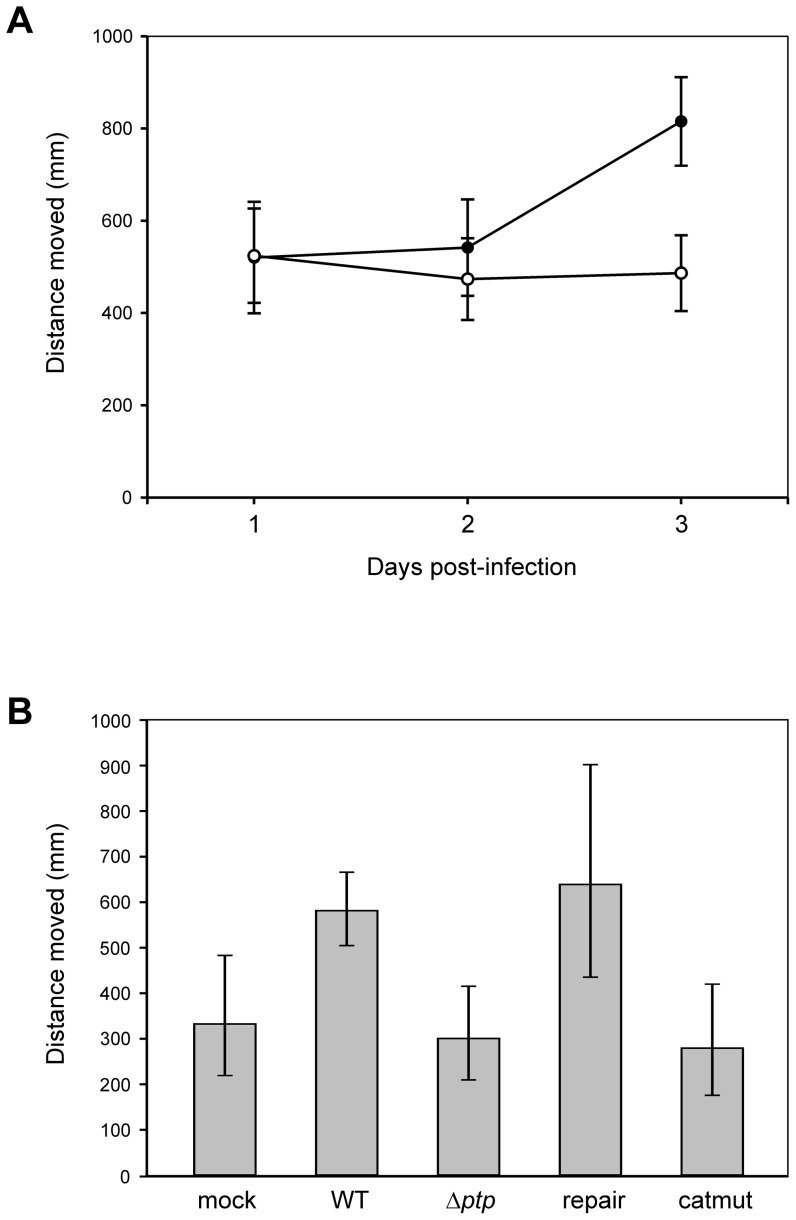
AcMNPV induces hyperactive behavior, and requires the phosphatase activity of the *ptp* encoded enzyme. (A) Estimated marginal means of distances moved (mm) by mock-infected (open circles) and WT-infected (closed circles) larvae in a 10-min interval at one, two and three dpi. Mock: n = 41, WT: n = 29. At three dpi, WT-infected larvae show higher activity than mock-infected ones (P<0.0001). Error bars represent 95% confidence intervals. (B) Estimated marginal means of distances moved (mm) by mock-, WT-, Δ*ptp*-, repair- and catmut-infected larvae in a 10-min interval at 3 dpi. Mock: n = 35, WT: n = 113, Δ*ptp*: n = 39, repair: n = 23, catmut: n = 24. WT-infected larvae show significantly higher activity than mock-, Δ*ptp-* and catmut-infected larvae (P = 0.0295, P = 0.001 and P = 0.0013, respectively). Error bars represent 95% confidence intervals.

To investigate whether AcMNPV induces hyperactive behavior in the permissive host *S. exigua*, movement assays were performed in an arena. Mock- and WT-infected larvae were tracked in the arena at one, two and three days post-infection (dpi) to check for differences in locomotor activity. A summary of the variation in the original data between different replicates, indicating the minimum and maximum distances measured for a certain treatment, is given in Table 2. Results showed that treatment (mock/WT) altered host mobility (*F*_(1,67.4)_ = 5.27; P = 0.0248), depending on the dpi (*F*_(2,65.7)_ = 9.25; P = 0.0003), and a highly significant interaction was found between treatment and dpi (treatment*day: *F*_(2,65.7)_ = 11.00; P<0.0001). On one and two dpi, distances moved were not significantly different between the two treatments (mock day 1: 524 mm, WT day 1: 520 mm, P = 1.000; mock day 2: 473 mm, WT day 2: 542 mm, P = 1.000) (Fig. 2A). However, at three dpi WT-infected larvae showed a significantly higher locomotor activity than mock-infected larvae (mock: 486 mm, WT: 815 mm, P<0.0001). Movement data later than three dpi were not included, as virus-infected larvae started to become moribund at four dpi.

**Table 2:** Variation between different replicates in movement assays of mock- and WT-infected larvae at one, two and three dpi.

		Distance moved (mm)		
Days post-infection (dpi)	Treatment	Minimum	Maximum	Mean ± SE
1 dpi	mock	501.1	532.5	516.8±51.45
	WT	494.8	540.7	520.1±60.07
2 dpi	mock	392.0	548.3	474.1±45.99
	WT	525.9	561.3	541.8±50.29
3 dpi	mock	481.0	503.6	492.6±42.68
	WT	803.3	830.0	815.3±45.72

Minimum, maximum and mean (± SE) distances moved of all replicates are displayed.

### Hyperactive behavior is induced by the AcMNPV ptp gene, but not by a mutant ptp gene encoding a catalytically inactive enzyme

Next, we examined a possible role for the *ptp* gene in the induction of the observed hyperactivity, by analyzing locomotor activity of mock-, WT-, Δ*ptp*, repair- and catmut-infected larvae at three dpi. Table 3 summarizes the variation in the original data between different replicates, indicating the minimum and maximum distances measured for all different treatments. As no interaction was detected between experiment and treatment (*F*_(5,228)_ = 0.17; P = 0.9724), this term was excluded from the model. After correction of the transformed data for between-experiment variation (*F*_(6,223)_ = 2.31; P = 0.0350), results showed a clear relation between virus treatment and larval activity (*F*_(4,223)_ = 7.73; P<0.0001). WT-infected larvae again showed a higher activity than uninfected control larvae (WT: 581 mm, mock: 333 mm, P = 0.0295), while Δ*ptp*-infected individuals moved a distance of 301 mm, which was significantly lower than WT-infected individuals (P = 0.001) (Fig. 2B). Infection with a repair virus, in which the *ptp* gene was placed back in the viral genome, restored the hyperactive phenotype observed in WT-infected larvae (639 mm, P = 1.000). Larvae infected with a catmut recombinant virus, carrying a catalytically mutated *ptp* gene, showed significantly lower activity than WT-infected larvae (279 mm, P = 0.0013).

**Table 3:** Variation between different replicates in movement assays of mock-, WT-, Δ*ptp*-, repair- and catmut-infected larvae at three dpi.

	Distance moved (mm)		
Treatment	Minimum	Maximum	Mean ± SE
mock	403.7	630.3	455.5±54.90
WT	575.1	937.6	687.2±44.55
Δ*ptp*	376.8	573.6	481.9±54.68
repair	663.2	686.4	674.3±107.07
catmut	265.6	267.1	266.3±39.05

Minimum, maximum and mean (± SE) distances moved of all replicates are displayed.

**Figure 3:**
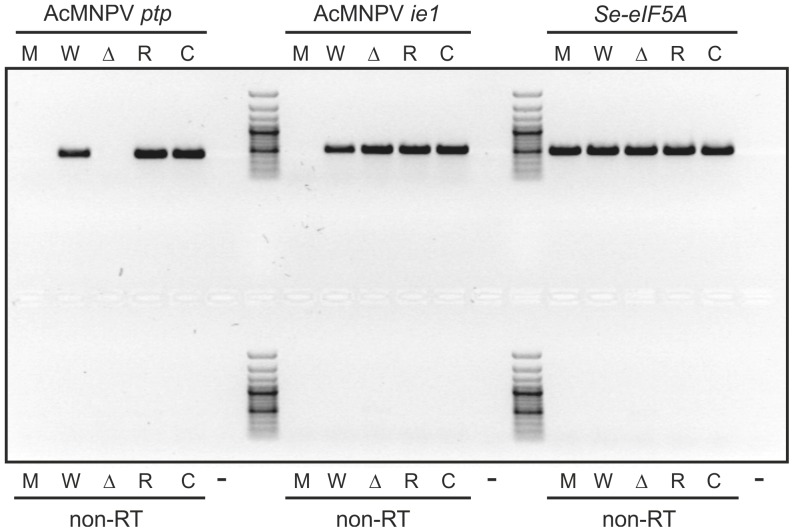
The AcMNPV *ptp* gene is expressed in the WT-, repair- and catmut-infected larvae. RT-PCR analysis on mock- (M), WT- (W), Δ*ptp*- (Δ), repair- (R) and catmut-infected (C) larvae. Expression of the AcMNPV *ptp* gene, the AcMNPV *ie1* gene and the host *Se*-*eIF5A* gene was analyzed. For each RT sample, a PCR without RT step (non-RT) was performed in parallel. For each primer pair, a no-template control was processed (-). The GeneRuler 100 bp ladder (Fermentas) was included in the agarose gel to estimate PCR fragment sizes.

To exclude the possibility that the observed behavioral phenotype of the catmut-infected larvae was due to inappropriate *ptp* expression, RT-PCR was performed on total RNA isolated from mock- or virus-infected single whole larvae at three dpi. The AcMNPV *ptp* gene was expressed in WT-, repair- and catmut-infected larvae, but expression was, as expected, absent in the mock- and Δ*ptp*-infected individuals (Fig. 3). The AcMNPV *ie1* gene, included as a control for virus infection, was expressed in all the virus-infected larvae, but not in the mock-infected ones. The *S. exigua eIF5A* gene, encoding eukaryotic translation initiation factor 5A [46], was included as a host control and showed expression in both mock- and virus-infected larvae.

### Phylogenetic analysis

**Figure 4:**
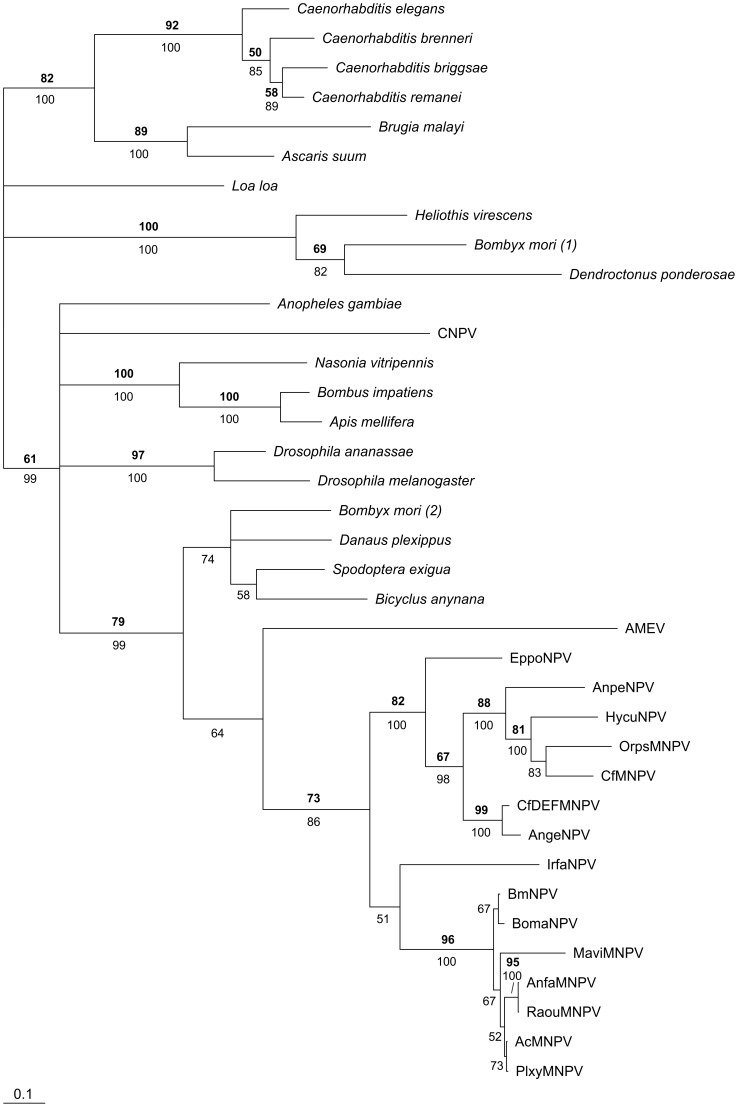
The baculovirus *ptp* gene was presumably acquired from a lepidopteran host by an ancestral NPV. Bayesian phylogeny of *ptp* nucleotide sequences from diverse invertebrate taxa. GenBank and Butterflybase/InsectaCentral accession numbers are given in Table S2. Numbers in bold indicate maximum likelihood bootstrap values based on 100 replicates, while plain numbers depict Bayesian posterior probabilities. Only values ≥50 are indicated for both analyses. The bar at the bottom indicates a branch length of 10% distance.

We investigated how *ptp* genes from baculoviruses and lepidopteran hosts were related using a phylogenetic approach, including all available invertebrate and baculovirus *ptp* sequences (Fig. 4). Two related poxvirus *ptp* sequences were included to see whether viral *ptp* homologues have a common origin. BLAST searches also revealed homologous vertebrate *ptp* sequences, but these were too distantly related to include in the phylogenetic analysis.

To better understand the origin of the baculovirus *ptp* gene, a baculovirus phylogeny was constructed using the baculovirus core gene *lef-8*, which encodes a subunit of the baculovirus RNA polymerase [47]. This highly conserved gene is very suitable for constructing a baculovirus phylogeny, distinguishing Alphabaculovirus group I NPVs, Alphabaculovirus group II NPVs and Betabaculoviruses (granuloviruses (GVs)) [48].

All the *ptp* sequences derived from baculovirus genomes form a monophyletic group, and form a well-supported clade with lepidopteran *ptp* sequences from *S. exigua*, *Bicyclus anynana*, *Danaus plexippus* and one of the two *ptp* sequences from *B. mori*, and with the *ptp* sequence from *Amsacta moorei* entomopoxvirus. As this clade localizes within the insect *ptp* sequences (using nematode *ptp* sequences as outgroup), a transfer of a *ptp* gene from a lepidopteran host to an ancestral baculovirus appears to be a likely evolutionary scenario (see below). Since the baculovirus and lepidopteran *ptp* sequences are not mixed in this phylogeny, this transfer presumably happened once, after which the gene was spread within the family *Baculoviridae*. The *ptp* from *A. moorei* entomopoxvirus (AMEV, *Poxviridae*) also localizes within this clade, although its exact position is unresolved. It could have been acquired from an (lepidopteran) insect host by transfer to an ancestral virus that gave rise to both the pox- and the baculoviruses, however, this scenario is unlikely as the *ptp* from the canarypoxvirus (CNPV, *Poxviridae*) is located elsewhere in the phylogeny. This does not support a common origin of *ptp* in viruses. Possibly, a transfer of *ptp* between an (ancestral) baculovirus and an (ancestral) entomopoxvirus occurred.

**Figure 5:**
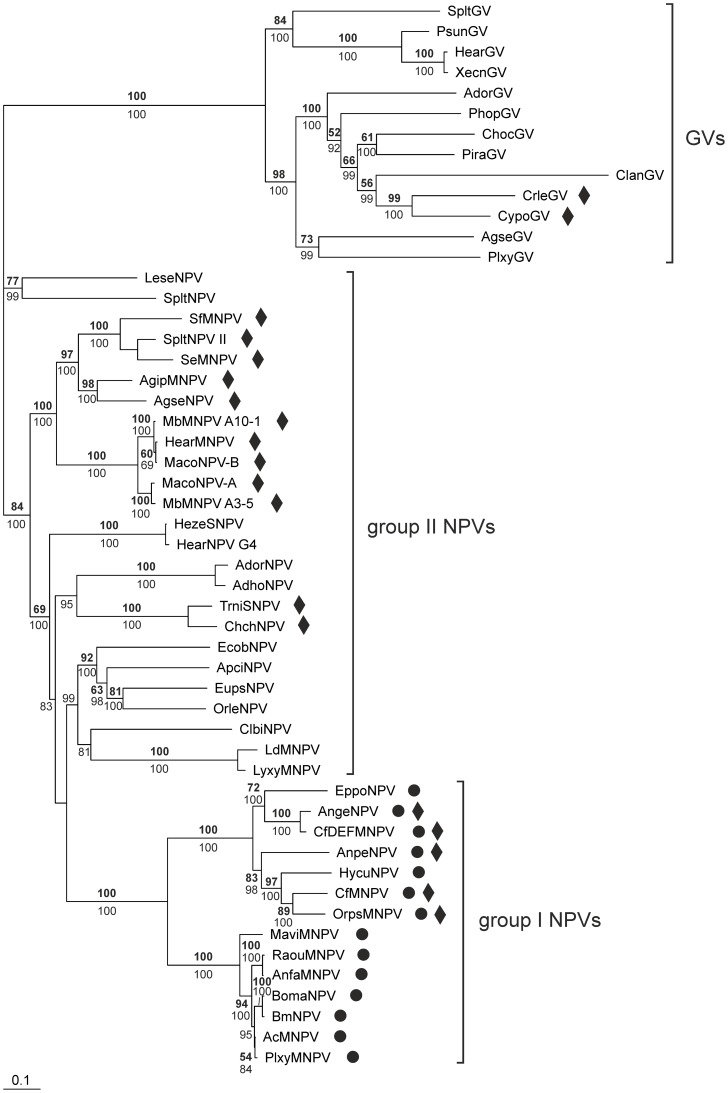
The *ptp* gene is present in all Alphabaculovirus group I NPVs. Bayesian phylogeny of baculoviruses based on the *lef-8* gene. GenBank accession numbers are given in Table S3. Numbers in bold indicate maximum likelihood bootstrap values based on 100 replicates, while plain numbers depict Bayesian posterior probabilities. Only values ≥50 are indicated for both analyses. The bar at the bottom indicates a branch length of 10% distance. Baculoviruses possessing a *ptp* gene are marked by a black dot, while baculoviruses possessing a *ptp2* gene are marked by a black diamond.

The baculovirus phylogeny (Fig. 5) shows that the *ptp* gene is present in all group I NPVs in the genus Alphabaculovirus, and absent in all Alphabaculovirus group II NPVs and Betabaculoviruses (GVs). The current hypothesis is that GVs and NPVs arose from a common ancestor, and that speciation of group I and II NPVs took place after divergence of GVs and NPVs [49]. The fact that only group I NPVs carry *ptp* indicates that the transfer of a lepidopteran host *ptp* to an ancestral baculovirus occurred after the group I and II NPVs diverged, or that the *ptp* gene was acquired before this divergence, and subsequently lost in (an ancestor of) group II NPVs and GVs. A transfer to an ancestral group I NPV baculovirus is the most parsimonious scenario.

In addition to the *B. mori ptp* sequence that is part of the lepidopteran clade showing high similarity to baculovirus *ptp*, *B. mori* also carries another *ptp* gene that is positioned in a different clade, together with *ptp* from *Heliothis virescens* (Lepidoptera) and *Dendroctonus ponderosae* (Coleoptera) (Fig. 4). The presence of this different *ptp* sequence within the *B. mori* genome could indicate an ancient gene duplication event, after which the different *ptp* copies diverged in sequence and, possibly, function. Alternatively, these two genes were acquired during two separate gene transfer events. Not all lepidopteran species carry two *ptp* genes: for *B. anynana* and *D. plexippus*, only one copy was found. Whether *S. exigua* and *H. virescens* carry two copies is unknown since no whole genome information is available for these organisms.

Besides the presence of *ptp* in group I NPVs, another gene encoding a putative PTP protein is present in several baculovirus genomes. This gene, named *ptp2*, is present in a number of group II NPVs, and in two GVs (Fig. 5).

## Discussion

Baculoviruses have long been known to alter behavior of their insect hosts [14], [15], [50], but the mechanisms underlying these behavioral changes are still poorly understood. The involvement of the viral *ptp* gene in hyperactivity has been shown for *B. mori* larvae infected with BmNPV in a study that has provided valuable first insights into baculovirus-induced behavioral changes [17]. However, it remained uncertain whether *B. mori*, which underwent massive artificial selection throughout its domestication, reflects a truly ecologically relevant model system to study such manipulative strategies. We used the baculovirus AcMNPV and its natural host *S. exigua* as a model system to study the degree of conservation of *ptp*-induced behavioral manipulation in lepidopteran hosts. We show that AcMNPV induces hyperactive behavior and that this behavioral change is absent when the catalytic site of the encoded phosphatase enzyme is mutated. These data, together with the finding that the *ptp* gene is well-conserved in a group of related baculoviruses (group I NPVs), provide solid evidence that *ptp*-induced hyperactivity represents an evolutionary conserved strategy to manipulate host behavior.

In the same study by Kamita *et al.* (2005) it was also shown that insertion of a *B. mori* host-derived *ptp* homolog in a BmNPVΔ*ptp* genome partially restored the hyperactive phenotype [17]. Our phylogenetic analysis suggests that the baculovirus *ptp* gene has a lepidopteran origin, indicating that an ancestral virus may have acquired this gene from its host in order to manipulate endogenous host pathways involved in behavior. Our analysis also demonstrates the presence of *ptp* in all group I NPV genomes sequenced so far. Besides its presence in baculoviruses, arthropods and nematodes, a *ptp* homolog was also found in the poxviruses AMEV and CNPV. To our knowledge no behavioral changes have been described for poxvirus-infected hosts so far.

An important issue to address is the ecological relevance of the observed increased host activity, *i.e.*, how could it contribute to virus transmission? Firstly, the increased movement of infected larvae ensures that progeny virions will be released over a larger surface, contributing to a higher chance of encountering a new host. Secondly, it is also thought that infected larvae start to ‘leak’ virus at a certain time after infection. Indeed, during our experiments, we occasionally observed larvae leaving a liquid trail, possibly containing virus particles, at three dpi (data not shown). Similar observations were reported during other studies [17]. Thirdly, increased activity in the field may also increase visibility to predator animals such as birds, which could serve as dispersal agent for the virus [50]. An alternative explanation might be that increased movement of infected larvae reflects an adaptive strategy of the host to prevent virus transmission to conspecifics, as it could result in removal of viral inoculum from the insect population [15].

Regarding the magnitude of the observed behavioral changes, it is interesting to note that in the studies performed with BmNPV-infected *B. mori* larvae, activity differences between WT- and mock-infected larvae were large (approximately 80-fold), while in our work an approximately 1.5-fold difference in activity between mock- and WT-infected individuals was observed. However, as discussed in the introduction, the *B. mori* insect strain used for the hyperactivity study by Kamita *et al.* (2005) displayed an exceptionally low level of endogenous activity [17]. In contrast, the *S. exigua* larvae used in our experiments were not selected for any particular behavioral trait (although selection pressures affecting behavioral traits in cultured insect populations cannot be excluded). Therefore, the differences in activity found in the current work may better reflect the natural situation. In our studies, mock-infected control larvae show a high endogenous activity, moving an average distance of 400–450 mm in 10 min. A 1.5-fold increase in distance moved of the observed population would over a 24 h time span lead to an approximately 40 meter difference in distance travelled between WT- and mock-infected larvae (excluding feeding and resting periods). For an efficient spread of virus particles over a large area such a difference could be a considerable factor.

Our findings seem to be in contrast with a recent study by Katsuma *et al.* (2012), reporting that in BmNPV-infected *B. mori* larvae PTP phosphatase activity was not required for the induction of hyperactivity. BmNPVΔ*ptp*-infected larvae showed reduced OB and BV production, and lower viral expression in many larval tissues, including the brain [18]. The authors hypothesize that this reduction of viral replication in the brain causes the behavioral phenotype of BmNPVΔ*ptp*-infected larvae. Although this is an interesting observation that could explain the fact that in cell culture the deletion of the AcMNPV *ptp* gene results in lower viral infectivity [23], it does not explain the mechanism by which this lower level of BmNPV viral expression in the host brain would contribute to altered behavior in *B. mori*. The difference in behavioral phenotype between the *ptp* catalytic mutant in the BmNPV/*B. mori* system and the AcMNPV/*S. exigua* system may imply that distinct mechanisms underlie hyperactivity in these two systems. Several studies have shown that AcMNPV PTP is associated with virions, similar to BmNPV PTP [51], [52]. Possibly, AcMNPV PTP functions both as a structural protein and a phosphatase. Minor variations in amino acid sequence (the proteins have 97% similarity) may play a role in this difference. Distinct mechanisms may also contribute to the difference in magnitude of the observed hyperactive phenotype.

In addition, there are several important differences in experimental setup between the studies that need to be considered. Firstly, *B. mori* larvae of the L5 stage were used in the study by Katsuma *et al.* (2012), while in the current study *S. exigua* L3 larvae were used. It is known from other studies that behavior, including locomotor activity, is influenced by developmental stage [15], [17], [50]. Furthermore, movement assays with *B. mori* were performed with groups of larvae, while in this study movement assays were performed with individual larvae. This difference in experimental setup could have important behavioral consequences. For solitary host species, such as the *S. exigua* L3 larvae used in this study (the L1 and L2 stage are gregarious, after which the larvae become solitary [53]), the motivation to move away from conspecifics could be high, while gregarious species may tend to remain together. Future research should provide more insight in whether these factors are of importance in the observed behavioral differences between the two studies.

The finding that in AcMNPV a *ptp* gene encoding a catalytically active PTP enzyme is needed for the induction of hyperactivity has important implications for understanding the molecular mechanisms underlying baculovirus-induced behavior. It suggests that AcMNPV PTP targets one or more phosphorylated substrate(s), either virus- or host-derived, which subsequently leads to the observed behavioral alteration. This opens up exciting new possibilities for further research to understand in more detail the mechanism of PTP-induced behavioral manipulation. For example, a differential transcriptome and (phospho-)proteome analysis could be employed in which WT- and Δ*ptp*-infected larvae are compared. Such experiments will shed light on possible host proteins and pathways affected by PTP expression. In addition, a host candidate-gene approach [54] could be followed in which host genes known to be involved in a behavioral phenotype in different insect species are investigated for their involvement in parasitic manipulation of behavior and their possible link with PTP.

Although the role of *ptp* in behavioral manipulation, despite possible variation in mechanism, appears to be conserved within group I NPVs, none of the group II NPVs or GVs carries a *ptp* gene (Fig. 5). Nevertheless, several studies indicate that group II NPVs may also alter host behavior. For example, Goulson (1997) reported that *Mamestra brassicae* larvae infected with *M. brassicae* nucleopolyhedrovirus (MbMNPV) showed higher locomotor activity in both laboratory and field experiments compared to uninfected individuals [15]. As the MbMNPV genome sequence has not been fully sequenced, we cannot exclude the possibility that MbMNPV carries a *ptp* gene, which may explain these behavioral changes. However, its position within the group II NPVs (Fig. 5), in which no baculovirus known so far contains a *ptp* gene, suggests that *ptp* is absent from its genome. This would imply that baculoviruses have developed different strategies to manipulate host behavior. Interestingly, MbMNPV, together with a subset of the group II NPVs and two GVs (Fig. 5), carries a *ptp2* gene encoding a protein that belongs to the same PTP protein family as AcMNPV PTP, although the similarity between the two proteins is very low (47% similarity over a 53 amino acid region). Whether this gene may have a similar function as *ptp* in manipulating host behavior is unknown.

Besides hyperactivity, baculoviruses are also known to alter climbing behavior of their insect hosts [15], [50]. A recent study showed involvement of the ecdysteroid UDP-glucosyltransferase (*egt*) gene from *L. dispar* multiple nucleopolyhedrovirus (LdMNPV) in the induction of climbing behavior in *L. dispar* larvae [16]. LdMNPV is a group II NPV that does not carry a *ptp* gene. Hyperactivity was not consistently observed in LdMNPV-infected *L. dispar* larvae (K. Hoover, unpublished results), possibly indicating that climbing and hyperactivity are two distinct behaviors induced by baculoviruses, for which different viral genes are responsible.

In addition to *ptp* and *egt*, other viral genes might affect larval behavior. Recently, Biernat *et al.* (2011) showed that a DNA repair protein (PHR2) encoded by the baculovirus *Chrysodeixis chalcites* nucleopolyhedrovirus (ChchNPV) can mimic the function of mammalian cryptochromes, essential regulators of the circadian clock [55]. It remains to be elucidated whether this protein can alter host circadian rhythm-related behavior, but this could indicate yet another strategy of baculoviruses to manipulate host insect behavior.

Understanding the molecular mechanisms underlying parasitic manipulation of host behavior, which is an example of the extended phenotype, is a fascinating new research field, in which knowledge from behavioral genetics, ecology, and parasitology is united. It provides invaluable information on the variety and complexity of host manipulation strategies, and on the evolutionary arms race between parasites and their hosts. The sophisticated ways employed by baculoviruses to manipulate host behavior provide an excellent starting point for understanding such underlying mechanisms.

## Supporting Information

Table S1 **Overview of primers used in this study.**
(DOCX)

Table S2 ***Protein tyrosine phosphatase*** **sequences used for phylogenetic analysis.**
(DOCX)

Table S3 ***Lef-8*** **sequences used for phylogenetic analysis.**
(DOCX)

## References

[1] Beckage NE (1997) Parasites and pathogens - Effects on host hormones and behavior. New York: Chapman and Hall.

[2] Moore J (2002) Parasites and the behavior of animals. Oxford: Oxford University Press.

[3] Lefèvre T, Adamo SA, Biron DG, Missé D, Hughes D, et al (2009) Invasion of the body snatchers: The diversity and evolution of manipulative strategies in host-parasite interactions. Advances in Parasitology 68: 45–83. 19289190 10.1016/S0065-308X(08)00603-9

[4] Dawkins R (1982) The extended phenotype. Oxford: Oxford University Press.

[5] Moore J (1995) The behavior of parasitized animals. Bioscience 45: 89–96.

[6] Combes C (1991) Ethological aspects of parasite transmission. American Naturalist 138: 866–880.

[7] Lefèvre T, Thomas F (2008) Behind the scene, something else is pulling the strings: Emphasizing parasitic manipulation in vector-borne diseases. Infection Genetics and Evolution 8: 504–519. 10.1016/j.meegid.2007.05.008 17588825

[8] Bennett KE, Hopper JE, Stuart MA, West M, Drolet BS (2008) Blood-feeding behavior of vesicular stomatitis virus infected *Culicoides sonorensis* (Diptera: Ceratopogonidae). Journal of Medical Entomology 45: 921–926. 18826036 10.1603/0022-2585(2008)45[921:bbovsv]2.0.co;2

[9] Luz PM, Lima-Camara TN, Bruno RV, de Castro MG, Sorgine MH, et al (2011) Potential impact of a presumed increase in the biting activity of dengue-virus-infected *Aedes aegypti* (Diptera: Culicidae) females on virus transmission dynamics. Mem Inst Oswaldo Cruz 106: 755–758. 22012232 10.1590/s0074-02762011000600017

[10] Lima-Camara TN, Bruno RV, Luz PM, Castro MG, Lourenço-de-Oliveira R, et al (2011) Dengue infection increases the locomotor activity of *Aedes aegypti* females. PLoS ONE 6. 10.1371/journal.pone.0017690 PMC3050906 21408119

[11] Platt KB, Linthicum KJ, Myint KSA, Innis BL, Lerdthusnee K, et al (1997) Impact of dengue virus infection on feeding behavior of *Aedes aegypti* . American Journal of Tropical Medicine and Hygiene 57: 119–125. 9288801 10.4269/ajtmh.1997.57.119

[12] Lefèvre T, Lebarbenchon C, Gauthier-Clerc M, Missé D, Poulin R, et al (2009) The ecological significance of manipulative parasites. Trends in Ecology & Evolution 24: 41–48. 19026461 10.1016/j.tree.2008.08.007

[13] Libersat F, Delago A, Gal R (2009) Manipulation of host behavior by parasitic insects and insect parasites. Annual Review of Entomology 54: 189–207. 10.1146/annurev.ento.54.110807.090556 19067631

[14] Hofmann O (1891) Die Schlaffsucht (Flacherie) der Nonne (*Liparis monacha*) nebst einem Anhang. Insektentötende Pilze mit besonderer Berücksichtigung der Nonne. P. Weber, Frankfurt.

[15] Goulson D (1997) Wipfelkrankheit: Modification of host behaviour during baculoviral infection. Oecologia 109: 219–228. 28307172 10.1007/s004420050076

[16] Hoover K, Grove M, Gardner M, Hughes DP, McNeil J, et al (2011) A gene for an extended phenotype. Science 333: 1401–1401. 21903803 10.1126/science.1209199

[17] Kamita SG, Nagasaka K, Chua JW, Shimada T, Mita K, et al (2005) A baculovirus-encoded protein tyrosine phosphatase gene induces enhanced locomotory activity in a lepidopteran host. Proceedings of the National Academy of Sciences of the United States of America 102: 2584–2589. 15699333 10.1073/pnas.0409457102 PMC548987

[18] Katsuma S, Koyano Y, Kang W, Kokusho R, Kamita SG, et al (2012) The baculovirus uses a captured host phosphatase to induce enhanced locomotory activity in host caterpillars. PLoS Pathog 8: e1002644. 22496662 10.1371/journal.ppat.1002644 PMC3320614

[19] Xia QY, Guo YR, Zhang Z, Li D, Xuan ZL, et al (2009) Complete resequencing of 40 genomes reveals domestication events and genes in silkworm (*Bombyx*). Science 326: 433–436. 19713493 10.1126/science.1176620 PMC3951477

[20] Goldsmith MR, Shimada T, Abe H (2005) The genetics and genomics of the silkworm, *Bombyx mori* . Annual Review of Entomology 50: 71–100. 10.1146/annurev.ento.50.071803.130456 15355234

[21] Shimada T (1999) Genetic mapping of virus resistances in *Bombyx mori* and *B. mandarina* . RIKEN Review 22: 68–72.

[22] Ayres MD, Howard SC, Kuzio J, Lopez-Ferber M, Possee RD (1994) The complete DNA sequence of *Autographa californica* nuclear polyhedrosis virus. Virology 202: 586–605. 8030224 10.1006/viro.1994.1380

[23] Li YH, Miller LK (1995) Properties of a baculovirus mutant defective in the protein phosphatase gene. Journal of Virology 69: 4533–4537. 7769718 10.1128/jvi.69.7.4533-4537.1995 PMC189200

[24] Tonks NK (2006) Protein tyrosine phosphatases: from genes, to function, to disease. Nature Reviews Molecular Cell Biology 7: 833–846. 17057753 10.1038/nrm2039

[25] Gross CH, Shuman S (1998) Characterization of a baculovirus-encoded RNA 5′-triphosphatase. Journal of Virology 72: 7057–7063. 9696798 10.1128/jvi.72.9.7057-7063.1998 PMC109926

[26] Takagi T, Taylor GS, Kusakabe T, Charbonneau H, Buratowski S (1998) A protein tyrosine phosphatase-like protein from baculovirus has RNA 5′-triphosphatase and diphosphatase activities. Proceedings of the National Academy of Sciences of the United States of America 95: 9808–9812. 9707557 10.1073/pnas.95.17.9808 PMC21418

[27] Sheng ZQ, Charbonneau H (1993) The baculovirus *Autographa californica* encodes a protein tyrosine phosphatase. Journal of Biological Chemistry 268: 4728–4733. 8444848

[28] Luckow VA, Lee SC, Barry GF, Olins PO (1993) Efficient generation of infectious recombinant baculoviruses by site-specific transposon-mediated insertion of foreign genes into a baculovirus genome propagated in *Escherichia coli* . Journal of Virology 67: 4566–4579. 8392598 10.1128/jvi.67.8.4566-4579.1993 PMC237841

[29] Groener A (1986) Specificity and safety of baculovirus. In: The biology of baculoviruses, pp. 177–202. Boca Raton: CRC Press.

[30] Smits PH, van de Vrie M, Vlak JM (1986) Oviposition of beet armyworm (Lepidoptera, Noctuidae) on greenhouse crops. Environmental Entomology 15: 1189–1191.

[31] Smith GE, Summers MD (1979) Restriction maps of 5 *Autographa californica* MNPV variants, *Trichoplusia ni* MNPV and *Galleria mellonella* MNPV DNAs with endonucleases SmaI, KpnI, BamHI, SacI, XhoI, and EcoRI. Journal of Virology 30: 828–838. 16789179 10.1128/jvi.30.3.828-838.1979 PMC353393

[32] Li Y, Guarino LA (2008) Roles of LEF-4 and PTP/BVP RNA triphosphatases in processing of baculovirus late mRNAs. Journal of Virology 82: 5573–5583. 18385232 10.1128/JVI.00058-08 PMC2395224

[33] Peng K, van Oers MM, Hu Z, van Lent JW, Vlak JM (2010) Baculovirus *per os* infectivity factors form a complex on the surface of occlusion-derived virus. Journal of Virology 84: 9497–9504. 20610731 10.1128/JVI.00812-10 PMC2937639

[34] Blissard GW (1996) Baculovirus-insect cell interactions. Cytotechnology 20: 73–93. 8987578 10.1007/BF00350390

[35] Kim D, Weaver RF (1993) Transcription mapping and functional analysis of the protein tyrosine serine phosphatase (PTPase) gene of the *Autographa californifica* nuclear polyhedrosis virus. Virology 195: 587–595. 8337833 10.1006/viro.1993.1410

[36] Martin JR (2003) Locomotor activity: a complex behavioural trait to unravel. Behavioural Processes 64: 145–160. 14556949 10.1016/s0376-6357(03)00132-3

[37] Box GEP, Cox DR (1964) An analysis of transformations. Journal of the Royal Statistical Society Series B-Statistical Methodology 26: 211–252.

[38] Papanicolaou A, Gebauer-Jung S, Blaxter ML, Owen McMillan W, Jiggins CD (2008) ButterflyBase: a platform for lepidopteran genomics. Nucleic Acids Res 36: D582–587. 17933781 10.1093/nar/gkm853 PMC2238913

[39] Pascual L, Jakubowska AK, Blanca JM, Cañizares J, Ferré J, et al (2012) The transcriptome of *Spodoptera exigua* larvae exposed to different types of microbes. Insect Biochem Mol Biol 42: 557–570. 22564783 10.1016/j.ibmb.2012.04.003

[40] Katoh K, Kuma K, Toh H, Miyata T (2005) MAFFT version 5: improvement in accuracy of multiple sequence alignment. Nucleic Acids Res 33: 511–518. 15661851 10.1093/nar/gki198 PMC548345

[41] Suyama M, Torrents D, Bork P (2006) PAL2NAL: robust conversion of protein sequence alignments into the corresponding codon alignments. Nucleic Acids Res 34: W609–612. 16845082 10.1093/nar/gkl315 PMC1538804

[42] Castresana J (2000) Selection of conserved blocks from multiple alignments for their use in phylogenetic analysis. Molecular biology and evolution 17: 540–552. 10742046 10.1093/oxfordjournals.molbev.a026334

[43] Swofford DL (2002) PAUP*, phylogenetic analysis using parsimony (*and other methods). Sinauer Associates, Sunderland.

[44] Ros VI, Fleming VM, Feil EJ, Breeuwer JA (2009) How diverse is the genus *Wolbachia*? Multiple-gene sequencing reveals a putatively new *Wolbachia* supergroup recovered from spider mites (Acari: Tetranychidae). Appl Environ Microbiol 75: 1036–1043. 19098217 10.1128/AEM.01109-08 PMC2643572

[45] Ronquist F, Huelsenbeck JP (2003) MrBayes 3: Bayesian phylogenetic inference under mixed models. Bioinformatics 19: 1572–1574. 12912839 10.1093/bioinformatics/btg180

[46] van Oers MM, van Marwijk M, Kwa MSG, Vlak JM, Thomas AAM (1999) Cloning and analysis of cDNAs encoding the hypusine-containing protein eIF5A of two lepidopteran insect species. Insect Molecular Biology 8: 531–538. 10620048 10.1046/j.1365-2583.1999.00148.x

[47] Guarino LA, Xu B, Jin JP, Dong W (1998) A virus-encoded RNA polymerase purified from baculovirus-infected cells. Journal of Virology 72: 7985–7991. 9733837 10.1128/jvi.72.10.7985-7991.1998 PMC110134

[48] Herniou EA, Jehle JA (2007) Baculovirus phylogeny and evolution. Curr Drug Targets 8: 1043–1050. 17979664 10.2174/138945007782151306

[49] Herniou EA, Olszewski JA, O'Reilly DR, Cory JS (2004) Ancient coevolution of baculoviruses and their insect hosts. Journal of Virology 78: 3244–3251. 15016845 10.1128/JVI.78.7.3244-3251.2004 PMC371050

[50] Vasconcelos SD, Cory JS, Wilson KR, Sait SM, Hails RS (1996) Modified behavior in baculovirus-infected lepidopteran larvae and its impact on the spatial distribution of inoculum. Biological Control 7: 299–306.

[51] Li YH, Miller LK (1995) Expression and localization of a baculovirus protein phosphatase. Journal of General Virology 76: 2941–2948. 8847498 10.1099/0022-1317-76-12-2941

[52] Wang RR, Deng F, Hou DH, Zhao Y, Guo L, et al (2010) Proteomics of the *Autographa californica* nucleopolyhedrovirus budded virions. Journal of Virology 84: 7233–7242. 20444894 10.1128/JVI.00040-10 PMC2898249

[53] Smits PH, van Velden MC, van de Vrie M, Vlak JM (1987) Feeding and dispersion of *Spodoptera exigua* larvae and its relevance for control with a nuclear polyhedrosis virus. Entomologia Experimentalis et Applicata 43: 73–80.

[54] Fitzpatrick MJ, Ben-Shahar Y, Smid HM, Vet LEM, Robinson GE, et al (2005) Candidate genes for behavioural ecology. Trends in Ecology & Evolution 20: 96–104. 16701349 10.1016/j.tree.2004.11.017

[55] Biernat MA, Eker AP, van Oers MM, Vlak JM, van der Horst GT, et al (2012) A baculovirus photolyase with DNA repair activity and circadian clock regulatory function. J Biol Rhythms 27: 3–11. 22306969 10.1177/0748730411429665

