# Supplementary material for: Protein Tyrosine Phosphatase-Induced Hyperactivity Is a Conserved Strategy of a Subset of BaculoViruses to Manipulate Lepidopteran Host Behavior
Source: PLoS One. 2012 Oct 15;7(10):e46933. doi: 10.1371/journal.pone.0046933 (PMC3471939; doi:10.1371/journal.pone.0046933)
Supplement: Table S1 — Overview of primers used in this study. (DOCX) [file pone.0046933.s001.docx]

**Table S1**. Overview of primers used in this study.

| **Primer number** | **Primer name** | **nt position on AcMNPV genome** | **Sequence** | **Function** |
| --- | --- | --- | --- | --- |
| 1 | *ptp* repair FW | 133865-133883 | GAG CCA TGG ACA TTA TCC CTC GAT TGT G | Amplify *hr1* sequence and insert NcoI site |
| 2 | *ptp* repair RV | 1059-1076 | GAG ATG CAT CGC TGG AAG AAG CGC AAC | Amplify *ptp* ORF and insert NsiI site |
| 3 | *ptp* catmut | 837-880 | GCA TGT TGG TGG GCG TGC ATG CCA CAC ACG GTA TTA ATC GCA CC | Create Cys to Ala mutation in catalytic site and insert SphI restriction site |
| 4 | poly-A-tail primer | - | GACCACGCGTATCGATGTCGACTTTTTTTTTTTTTTTV | Production of cDNA |
| 5 | Ac-*ptp* FW | 526-548 | CAACTATTTACAATGCGGCCAAG | RT-PCR on AcMNPV *ptp* |
| 6 | Ac-*ptp* RV | 926-945 | CTATCTATGGCTTCCTGCGG | RT-PCR on AcMNPV *ptp* |
| 7 | Ac-*ie1* FW | 128212-128232 | TAAGAATTCGTTGGGCGAAAGAAAATGT | RT-PCR on AcMNPV *ie1* |
| 8 | Ac-*ie1* RV | 128702-128724 | TAAAAGCTTCGCCAGAAATCCAATAAACT | RT-PCR on AcMNPV *ie1* |
| 9 | *Se*- *eIF5A* | - | GCCATGGCTGACATCGAGGATAC | RT-PCR on *S. exigua* (host) *eIF5A* |
| 10 | *Se*- *eIF5A* | - | GCGGTACCGGTTTATTTGTCGAGAGC | RT-PCR on *S. exigua* (host) *eIF5A* |
