# Supplementary material for: Protein Tyrosine Phosphatase-Induced Hyperactivity Is a Conserved Strategy of a Subset of BaculoViruses to Manipulate Lepidopteran Host Behavior
Source: PLoS One. 2012 Oct 15;7(10):e46933. doi: 10.1371/journal.pone.0046933 (PMC3471939; doi:10.1371/journal.pone.0046933)
Supplement: Table S2 — Protein tyrosine phosphatase sequences used for phylogenetic analysis. (DOCX) [file pone.0046933.s002.docx]

**Table S2**. *Protein tyrosine phosphatase* sequences used for phylogenetic analysis.

| **Group/phylum** | **Virus family/animal order** | **Species name** | **GenBank accession no.** |
| --- | --- | --- | --- |
| Viruses | *Baculoviridae* | *Anagrapha falcifera* MNPV (AnfaMNPV) | AY706539 (partial) |
|  | *Baculoviridae* | *Antheraea pernyi* NPV (AnpeNPV) | DQ486030 |
|  | *Baculoviridae* | *Anticarsia gemmatalis* NPV (AngeNPV) | DQ813662 |
|  | *Baculoviridae* | *Autographa californica* NPV (AcMNPV) | L22858 |
|  | *Baculoviridae* | *Bombyx mandarina* NPV (BomaNPV) | FJ882854 |
|  | *Baculoviridae* | *Bombyx mori* NPV (BmNPV) | L33180 |
|  | *Baculoviridae* | *Choristoneura fumiferana* DEF MNPV (CfDEFMNPV) | AY327402 |
|  | *Baculoviridae* | *Choristoneura fumiferana* MNPV (CfMNPV) | AF512031 |
|  | *Baculoviridae* | *Epiphyas postvittana* NPV (EppoNPV) | AY043265 |
|  | *Baculoviridae* | *Hyphantria cunea* NPV (HycuNPV) | AP009046 |
|  | *Baculoviridae* | *Iragoides fasciata* NPV (IrfaNPV) | FJ362523 |
|  | *Baculoviridae* | *Maruca vitrata* MNPV (MaviMNPV) | EF125867 |
|  | *Baculoviridae* | *Orgyia pseudotugata* MNPV (OrpsMNPV) | U75930 |
|  | *Baculoviridae* | *Plutella xylostella* MNPV (PlxyMNPV) | DQ457003 |
|  | *Baculoviridae* | *Rachiplusia ou* MNPV (RaouMNPV) | AY145471 |
|  | *Poxviridae* | *Amsacta moorei* entomopoxvirus (AMEV) | NC_002520 |
|  | *Poxviridae* | Canarypoxvirus (CNPV) | NC_005309 |
| Arthropoda | Diptera | *Anopheles gambiae* | XM_309494 |
|  | Diptera | *Drosophila ananassae* | XM_001960507 |
|  | Diptera | *Drosophila melanogaster* | NM_136852 |
|  | Coleoptera | *Dendroctonus ponderosae* | GT390125* |
|  | Hymenoptera | *Apis mellifera* | XM_001120896 |
|  | Hymenoptera | *Bombus impatiens* | XM_003489572 |
|  | Hymenoptera | *Nasonia vitripennis* | XM_003423942 |
|  | Lepidoptera | *Bicyclus anynana* | GE688573* |
|  | Lepidoptera | *Bombyx mori (1)* | BY938310* |
|  | Lepidoptera | *Bombyx mori (2)* | NM_001043503 |
|  | Lepidoptera | *Danaus plexippus* | EY272595* |
|  | Lepidoptera | *Heliothis virescens* | GT199458* |
|  | Lepidoptera | *Spodoptera exigua* | Courtesy of S. Herrero |
| Nematoda | Ascaridida | *Ascaris suum* | JI180758 |
|  | Rhabditida | *Caenorhabditis brenneri* | GL379872 |
|  | Rhabditida | *Caenorhabditis briggsae* | XM_002629654 |
|  | Rhabditida | *Caenorhabditis elegans* | NM_063558 |
|  | Rhabditida | *Caenorhabditis remanei* | XM_003097075 |
|  | Spirurida | *Brugia malayi* | XM_001897267 |
|  | Spirurida | *Loa loa* | ADBU01002848 |

GV, granulovirus; NPV, nucleopolyhedrovirus; SNPV, single nucleopolyhedrovirus; MNPV, multiple nucleopolyhedrovirus; DEF, defective

* Butterflybase/InsectaCentral: <http://insectacentral.org/>
