# Supplementary material for: Protein Tyrosine Phosphatase-Induced Hyperactivity Is a Conserved Strategy of a Subset of BaculoViruses to Manipulate Lepidopteran Host Behavior
Source: PLoS One. 2012 Oct 15;7(10):e46933. doi: 10.1371/journal.pone.0046933 (PMC3471939; doi:10.1371/journal.pone.0046933)
Supplement: Table S3 — Lef-8 sequences used for phylogenetic analysis. (DOCX) [file pone.0046933.s003.docx]

Table S3. *Lef-8* sequences used for phylogenetic analysis.

| **Virus name** | **Abbreviation** | **GenBank accession no.** |
| --- | --- | --- |
| *Adoxophyes honmai* NPV | AdhoNPV | AP006270 |
| *Adoxophyes orana* GV | AdorGV | AF547984 |
| *Adoxophyes orana* NPV | AdorNPV | EU591746 |
| *Agrotis ipsilon* MNPV | AgipMNPV | EU839994 |
| *Agrotis segetum* GV | AgseGV | AY522332 |
| *Agrotis segetum* NPV | AgseNPV | DQ123841 |
| *Anagrapha falcifera* MNPV | AnfaMNPV | AY706539 (partial) |
| *Antheraea pernyi* NPV | AnpeNPV | DQ486030 |
| *Anticarsia gemmatalis* NPV | AngeNPV | DQ813662 |
| *Apocheima cinerarius* NPV | ApciNPV | FJ914221 |
| *Autographa californica* NPV | AcMNPV | L22858 |
| *Bombyx mandarina* NPV | BomaNPV | FJ882854 |
| *Bombyx mori* NPV | BmNPV | L33180 |
| *Choristoneura fumiferana* DEF MNPV | CfDEFMNPV | AY327402 |
| *Choristoneura fumiferana* MNPV | CfMNPV | AF512031 |
| *Choristoneura occidentalis* GV | ChocGV | DQ333351 |
| *Chrysodeixis chalcites* NPV | ChchNPV | AY864330 |
| *Clanis bilineata* NPV | ClbiNPV | DQ504428 |
| *Clostera anachoreta* GV | ClanGV | HQ116624 |
| *Cryptophlebia leucotreta* GV | CrleGV | AY229987 |
| *Cydia pomonella* GV | CypoGV | U53466 |
| *Ecotropis obliqua* NPV | EcobNPV | DQ837165 |
| *Epiphyas postvittana* NPV | EppoNPV | AY043265 |
| *Euproctis pseudoconspersa* NPV | EupsNPV | FJ227128 |
| *Helicoverpa armigera* GV | HearGV | EU255577 |
| *Helicoverpa armigera* MNPV | HearMNPV | EU730893 |
| *Helicoverpa armigera* NPV G4 | HearNPV G4 | AF271059 |
| *Helicoverpa zea* SNPV | HezeSNPV | AF334030 |
| *Hyphantria cunea* NPV | HycuNPV | AP009046 |
| *Leucania separata* NPV | LeseNPV | AY394490 |
| *Lymantria dispar* MNPV | LdMNPV | AF081810 |
| *Lymantria xylina* MNPV | LyxyMNPV | GQ202541 |
| *Mamestra brassicae* MNPV A3-5 | MbMNPV A3-5 | AY706567 (A3-5, partial) |
| *Mamestra brassicae* MNPV A10-1 | MbMNPV A10-1 | AY706566 (A10-1, partial) |
| *Mamestra configurata* NPV-A | MacoNPV-A | U59461 |
| *Mamestra configurata* NPV-B | MacoNPV-B | AY126275 |
| *Maruca vitrata* MNPV | MaviMNPV | EF125867 |
| *Orgyia leucostigma* NPV | OrleNPV | EU309041 |
| *Orgyia pseudotsugata* MNPV | OrpsMNPV | U75930 |
| *Phthorimaea operculella* GV | PhopGV | AF499596 |
| *Pieris rapae* GV | PiraGV | GQ884143 |
| *Plutella xylostella* GV | PlxyGV | AF270937 |
| *Plutella xylostella* MNPV | PlxyMNPV | DQ457003 |
| *Pseudaletia unipuncta* GV | PsunGV | EU678671 |
| *Rachiplusia ou* MNPV | RaouMNPV | AY145471 |
| *Spodoptera exigua* MNPV | SeMNPV | AF169823 |
| *Spodoptera frugiperda* MNPV | SfMNPV | EF035042 |
| *Spodoptera litura* GV | SpltGV | DQ288858 |
| *Spodoptera litura* NPV | SpltNPV | AF325155 |
| *Spodoptera litura* NPV II | SpltNPV II | EU780426 |
| *Trichoplusia ni* SNPV | TrniSNPV | DQ017380 |
| *Xestia c-nigrum* GV | XecnGV | AF162221 |

GV, granulovirus; NPV, nucleopolyhedrovirus; SNPV, single nucleopolyhedrovirus; MNPV, multiple nucleopolyhedrovirus; DEF, defective
